# Doing Aging Research Together: Innovative Perspectives on Participatory Approaches

**DOI:** 10.1093/geroni/igab046.1503

**Published:** 2021-12-17

**Authors:** Anna Wanka, Anna Urbaniak

## Abstract

The symposium aims to take a closer look at what it means to involve older participants in ageing research - beyond the role of research subjects. By discussing projects that deploy different participatory approaches we investigate the manifold ways in which older adults can become co-creators of the research process. We do so comparing such approaches in different domains, with different outcomes and in different stages of the research process. Consequently, this symposium (1) looks at the research process through the lens of benefits and challenges resulting from involving older adults as co-creators; (2) showcases projects across different domains and different jurisdictions that applied participatory approach in ageing research to discuss benefits and challenges, and (3) advances scientific insights into participatory approaches involving older adults. After an introductory contribution outlining theories, concepts and developments of participatory approaches in ageing research, we present insights from three empirical studies in different cultural and thematic settings. In our first presentation, Anna Wanka and Anna Urbaniak open the symposium by presenting an overview of participatory approaches that involve older adults. In the first empirical presentation, Julia Nolte and Hamid Turker discuss the process of involving older adults in data analysis and therefor present data from the US. In the third presentation, Lillian Hunn highlights how the recent COVID-19 pandemic impacted patient involvement in research in Canada. Finally, Anna Urbaniak discusses the process of planning participatory research with hard to reach population among older adults in Austria, namely those who are socially excluded.

